# Protein–protein interaction network of *E. coli* K‐12 has significant high‐dimensional cavities: new insights from algebraic topological studies

**DOI:** 10.1002/2211-5463.13437

**Published:** 2022-06-16

**Authors:** Xiao‐yan Xue, Zhou Chen, Yue Hu, Dan Nie, Hui Zhao, Xing‐gang Mao

**Affiliations:** ^1^ 12644 Department of Pharmacology School of Pharmacy Fourth Military Medical University Xi'an China; ^2^ Department of Neurosurgery Xijing Hospital Fourth Military Medical University Xi'an China

**Keywords:** Betti number, complex system, drug resistance, higher‐order interactions, Network Science, simplex

## Abstract

As a model system, *Escherichia coli* has been used to study various life processes. A dramatic paradigm shift has occurred in recent years, with the study of single proteins moving toward the study of dynamically interacting proteins, especially protein–protein interaction (PPI) networks. However, despite the importance of PPI networks, little is known about the intrinsic nature of the network structure, especially high‐dimensional topological properties. By introducing general hypergeometric distribution, we reconstruct a statistically reliable combined PPI network of *E. coli* (*E. coli*‐PPI‐Network) from several datasets. Unlike traditional graph analysis, algebraic topology was introduced to analyze the topological structures of the *E. coli*‐PPI‐Network, including high‐dimensional cavities and cycles. Random networks with the same node and edge number (RandomNet) or scale‐free networks with the same degree distribution (RandomNet‐SameDD) were produced as controls. We discovered that the *E. coli*‐PPI‐Network had special algebraic typological structures, exhibiting more high‐dimensional cavities and cycles, compared to RandomNets or, importantly, RandomNet‐SameDD. Based on these results, we defined degree of involved *q*‐dimensional cycles of proteins (*q*‐DC_protein_) in the network, a novel concept that relies on the integral structure of the network and is different from traditional node degree or hubs. Finally, top proteins ranked by their 1‐DC_protein_ were identified (such as gmhB, rpoA, rplB, rpsF and yfgB). In conclusion, by introducing mathematical and computer technologies, we discovered novel algebraic topological properties of the *E. coli*‐PPI‐Network, which has special high‐dimensional cavities and cycles, and thereby revealed certain intrinsic rules of information flow underlining bacteria biology.

## Graphical abstract

We studied the high‐dimensional topological properties of protein–protein interaction network of *Escherichia* *coli* (*E. coli*‐PPI‐Network), which is performed on a statistically‐reliable combined PPI network reconstructed by using general hypergeometric distribution. Different from traditional graph analysis, we found that *E. coli*‐PPI‐Network had special algebraic typological structures compared to their corresponding random networks. These results revealed certain intrinsic rules of information flow underlining bacteria biology.

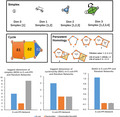

**Abbreviations**
BDC: biggest dimension of cavity that is the maximal dimension for all cavities
BDS: biggest dimension of simplex
Betti_q_
: *q*‐dimension Betti numbers
CI: confidential interval
D_protein_
: degree of proteins in a network
*E. coli*‐PPI‐Network: protein–protein interaction network of *E. coli*
GHGD: general hypergeometric distribution
HDATS: high‐dimensional algebraic topological structures
HG: homology group
PPI: protein–protein interaction
*q*‐DC_protein_
: degree of involved *q*‐dimensional cycles of proteins
RandomNet: Random networks with the same node and edge number of a network
RandomNet‐SameDD: scale‐free networks with the same degree distribution of a network

A dramatic paradigm shift has occurred in recent years, from traditional study of single proteins to study of group of dynamically interacting proteins, which formed complex protein–protein interaction (PPI) networks [1, 2, 3, 4, 5, 6, 7]. Because bacterial PPI networks are less complex but more diverse than their plant or animal counterparts, it is important to study their structures and properties to reveal principles of network organization [8]. *Escherichia coli* K‐12, as a representative strain of Gram‐negative bacterium in a central position within the microbial research community, is one of the best characterized organisms and has served as a model system to study many aspects of bacterial physiology. However, despite increasingly advances in both theoretical and technical approaches of mapping the protein interactions [9, 10, 11], little is known about the organizational principles of the PPI networks, mainly as a result of a lack of feasible approaches, because analysis of PPI networks relies on interdisciplinary areas including biology, computer science, and mathematics. Traditional graph approaches have been developed to study the topological features of PPI networks [12], including degree, clustering coefficient, betweenness, closeness, assortativity, shortest path between two nodes, and so on. However, only very limited intrinsic properties of the PPI networks were revealed by these parameters, and most studies focused on the degree of the proteins, especially those with large degrees, namely hub proteins.

As complex metric independent geometry objects, PPI networks exhibited obvious high‐dimensional abstract topological structures that are important for information transduction. However, for very long time, these high‐dimensional topological structures and their biological significance were not explored. As a young field in mathematics, algebraic topology deals with high‐dimensional metric independent geometry objects by taking advantage of modern algebra, which quantitatively describes the intrinsic features of high‐dimensional algebraic topological structures (HDATS) of networks, such as simplexes, cavities, and cycles [13, 14]. In addition, remarkably, the results of algebraic topology analysis also revealed rules of information flow in high‐dimensional cycles, which cannot be described by traditional graph analysis [15].

Here, by integrating biology study, mathematical theory, and computer science, we first investigated the algebraic topological structures of PPI networks of *E. coli* (*E. coli*‐PPI‐Network) and discovered that *E. coli*‐PPI‐Network contained significant HDATS, which is significantly different from random networks and corresponding scale‐free networks [16]. Our results not only revealed novel properties of the *E. coli*‐PPI‐Network in an integrated global perspective, but also provided novel approaches to find potential therapeutic targets which have critical impact on the essential functions of bacteria, such as survival, drug resistance, and so on.

## Methods

### Identification of statistically significant PPI interactions from several datasets

To get a reliable PPI interactions set of *E. coli*, we searched the Pubmed with key words ‘protein protein interaction network’, ‘*Escherichia coli*’, and ‘K‐12’, and read the relevant papers to find highly reliable PPI results that are validated by experimental and theoretical approaches. At last, three datasets were used for our analysis, comprising those of Arifuzzaman *et al*. [17], Hu *et al*. [18], and Rajagopala *et al*. Considering the great variation of high‐throughput data, we used overlapped data in the three datasets to get most reliable PPI interactions, by taking advantages of the general hypergeometric distribution (GHGD) [19]. Different from our previous paper, here, we used the interaction rather than the nodes of the networks as the overlapped elements in the GHGD analysis. The GHGD was used because, if we use the PPIs overlapped in all the three datasets, then we only get a few PPIs (in total, 37 interactions were overlapped in all of the three datasets) and would lose too many PPIs (the false negative is too high). However, if we use all the PPIs in the three files, the result would contain too many false positives. By using the formulas of mathematical expectations and variances of the GHGD, the 95% confidence interval (CI) of the GHGD can be estimated with Chebyshev's inequality, which gives an upper bound of number of randomly overlapped elements (random_Up_PPIs). Then, the number of statistically significant overlapped PPIs (sig_PPIs) can be deduced by sig_PPIs = observed_PPIs − random_Up_PPIs.

### Calculation of homology group (HG) and Betti numbers

**Fig. 1:**
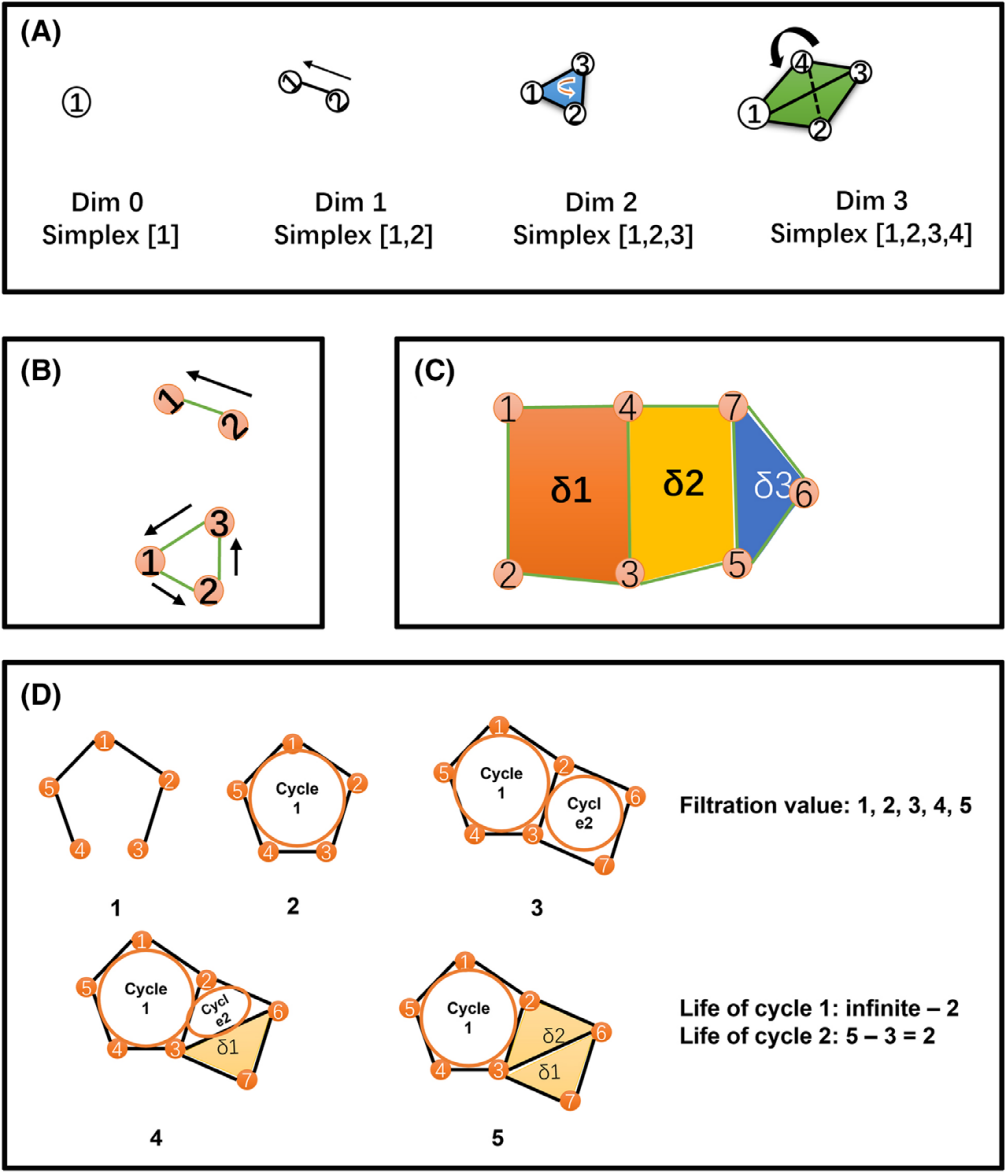
Schematic images illustrating the key concepts in algebraic topology and persistence homology. (A) Simplexes for different dimensions. (B) Calculation of boundary maps. Boundary map for Dim 1 Simplex ∂[1,2] = [2] − [1], and for Dim 2 Simplex ∂[1,2,3] = [1,2] + [2,3] + [3,1]. Furthermore, the boundary of the ‘Dim 2 Simplex Boundary’ is, ∂∂[1,2,3] = ∂[1,2] + ∂[2,3] + ∂[3,1] = [2] − [1] + [3] − [2] + [1] − [3] = 0. (C) Dim1 cycles (or cavities). There are two classes of Dim 1 cycles: **(1) δ1[1,2,3,4]; (2) δ2[3,5,7,4]** or **[3,5,6,7,4]**, these two are equal to each other, because they enclosed a same ‘cavity’ (yellow part). The two cavities are filled with orange and yellow colors respectively. Note that **δ3[5,6,7]** is a simplex but not a cycle. (D) Process of persistence homology. An example of filtration which starts at value 1 and ends at value 5. Each image represents a filtration step and is assigned with a value. At value 1, there are five dim 0 simplexes (that are points, [1], [2], [3], [4], [5]) and four dim 1 simplexes (that are edges, [1,2], [2,3], [4,5], [1,5]). At value 2, one more dim 1 simplex [3,4] is added into the complex and thus a dim 1 cycle (cycle 1) is formed; hence, the life of this dim 1 cycle starts at value 2. At value 3, two more dim 0 simplexes ([6], [7]) and three more dim 1 simplexes ([2,6], [3,7], [6,7]) are added and another dim 1 cycle (cycle 2) is formed for which life starts at value 3. At value 4, a dim 2 simplex δ1 ([3,6,7]) is added, and the two dim 1 cycles are persisted. At value 5, another dim 2 simplex δ2 ([2,3,6]) is added and thus cycle 2 is disappeared whose life ended at value 5, whereas cycle 1 still persisted. Therefore, there are totally two dim 1 cycles, and the life length of cycle 1 is infinite – 2 (here infinite indicates the cycle persists longer than the observed filtration values), while the life of cycle 2 is 5 − 3 = 2.

The definition of simplex, simplicial complex, chain, cavity, cycle, HG, and Betti numbers have been described in detail previously [14, 15]. Here, a simplicial complex *K* was made up of vertices and simplexes. A *p*‐dimensional simplex (*p*‐simplex) is defined as collections of (*p* + 1) full connected vertices. For example, a point is a 0‐simplex, a set of two points that are connected to each other is a 1‐simplex, and a set of three points that are connected to each other is a 2‐simplex, and so on. (Fig. 1A). It should be noted that a *p*‐simplex is a *p*‐dimension object. Any subset of the vertices of one simplex is a ‘face’ of the simplex. Specifically, the edges are 1‐dimensional faces of a simplex. To make the simplexes be calculable with algebraic approaches, assign a value (or an element) in a *group* for each of the *p*‐simplex (coefficient *group*; here *group* is a conception in algebra, which is defined as follows: a group is an algebraic structure consisting of a set of elements equipped with a binary operation that combines any two elements to form a third element. To be a group, this operation must satisfy four conditions called the group axioms: closure, associativity, identity, and invertibility). An operation for the *p*‐simplex was defined as the same operation of its corresponding element in the *group*. Therefore, a finite number of *p*‐simplex with the above‐defined operation formed a *chain* with *p*‐dimension (*p*‐chain). For example, for a set of *p*‐simplexes, *s*_1_, *s*_2_, …, *s*_l_, each *s*_i_ can be represented by its vertices: $\sigma_{k}=\sigma \left[v_{i_{1}},v_{i_{2}},\cdots ,\;v_{i_{p}}\;\right]$, and the p‐chain is:

$$C_{p}\left(K\right)=\sum_{i=0}^{l}\sigma \left[v_{i_{1}},v_{i_{2}\;}\cdots v_{i_{p}}\right].$$

Therefore, each *p*‐chain has a value belonging to the group. All of the *p*‐chains with the above‐defined operation form a group C_p_(*K*).

In the following description, group, map, image, kernel, and rank are all terminologies of group theory in modern algebra.

The simplex can be oriented according to the order of the vertices. Next, define the boundary *map* for each simplex *C*_p_(*K*) → *C*_p − 1_(*K*) ($\hat{v_{i_{j}}}$ indicates omission of the vertex $v_{i_{j}}$):

$$\partial \sigma \left[v_{i_{1}},v_{i_{2}\;},\cdots ,v_{i_{k}}\;\right]=\sum_{j=0}^{k}{\left(-1\right)}^{j}\sigma \left[v_{i_{1}},v_{i_{2}}\cdots \hat{v_{i_{j}}}\cdots v_{i_{k}}\right].$$

It is clear that the boundary map ∂ transforms a *p*‐simplex to a (*p* − 1)‐simplex, and the result (*p* − 1)‐simplexes are denoted as a boundary for the p‐simplex. For a given *p*, the *image* from a upper dimension ∂_p + 1_ (*C*_p + 1_(*K*)) (im∂_p + 1_, that is boundaries of the p + 1 chains) is a subgroup of the *C*_p_(*K*), and the *kernel* of the ∂_p_ (*C*_p_(*K*)) [ker∂_p_, closed chains in the *C*_p_(*K*), which are termed as cycles] is also a subgroup of *C*_p_(*K*). Here, a *p*‐dimensional cycle (*p*‐cycle) is a ‘closed *p*‐chain’, that is, all the p‐simplexes constituting the *p*‐cycle have a 0 value in the above‐defined operation. In addition, considering that ∂_p + 1_ ∂_p_ = 0, any boundary from an upper dimension is a cycle, and therefore the im∂_p + 1_ is a subgroup of ker∂_p_ (Fig. 1B). Based on these definitions, the homology group for dimension *q*, *H*_q_(*K*), is defined as the quotient group:

$$H_{p}\left(K\right)=\frac{\ker\partial_{p}}{\mathrm{im}\partial_{p+1}}.$$

Behind the highly abstract definition process of the homology group, the *H*_p_(*K*) has special geometric meanings. For finite simplicial complexes, which is the main topic for the present PPI networks, the *H*_p_(*K*) is a finitely generated Abel group, and the rank of the group is called Betti number, Betti_q_. Intuitively, betti_0_ indicates the number of connected graphs, betti_1_ indicates the number of 1‐dimensional cavities, whereas betti_2_ indicates the number of 2‐dimensional cavities (geometrical structures similar to hollow spherical structures). As defined above, a *q*‐cavity is enclosed by an equivalent class of *q*‐cycles. Therefore, 1‐cycles can be viewed as traditional rings (Fig. 1C), whereas 2‐cycles can be viewed as the surface of a ball, but not containing the inside. It should be noted that, for the cycles, each cycle actually represents a class of cycles which are equal to each other based on the calculation of quotient group (e.g. in Fig. 1C, the two cycles, 3‐4‐7‐5 and 3‐4‐7‐6‐5, are equal to each other). In the present study, we would focus on the minimal cycles (e.g. in Fig. 1C, we would use the cycle 3‐4‐7‐5, but not 3‐4‐7‐6‐5). However, for cycle involvement of each node, to avoid loss of cycles a node participates, the equivalent cycles are used to represent a class of cycles. For example, in Fig. 1C, the nodes (‘3’, ‘4’, ‘7’, ‘5’, and ‘6’) are all involved in a same class of cycle which enclosed the same ‘cavity’ δ2. In the present study, the *Z*/*Z*2 *group* (a 2‐order cyclic group; here *Z* is an abelian group consisted of all integers in the operation addition ‘+’; *Z*2 is the group consisted of all even integers; *Z*/*Z*2 is the quotient group of *Z* and *Z*2, which is a 2‐order cyclic group containing two elements) was used as the coefficients *group*. Calculation of Betti numbers was performed using jplex [20].

For Betti curve analysis, a technique of persistent homology is used, as described previously. Briefly, there is a weight between two nodes for each edge, and larger weight values implied more reliable link between the two genes. Therefore, the network would be constructed by adding edges one by one, according to the rank of their weight values. This would produce a series of growing networks, which formed a *filtration*. In each step, Betti values were calculated, and at last, a series of Betti values were produced (Fig. 1D). Then, the number of growing edges versus the corresponding Betti values formed the Betti curves.

### Construction of random networks

To get topological features of random networks (RandomNet), networks with the defined nodes and edges were constructed. One edge was added in each step, until the total number of edges reached to the defined amount. Then, the above algebraic topological and PH analysis was performed to get betti number in each dimension. To get the distribution properties of the topological features of RandomNet, 1000 random networks samples were produced, and the Betti numbers were calculated with the above process, and the statistical distribution of these parameters was established.

To produce RandomNet‐SameDD, we utilized the Havel–Hakimi theorem. The Havel–Hakimi theorem was used to determine whether a degree sequence can form a graph. We used the reserve step of the Havel–Hakimi determination and added one edge each step randomly. Then, the degree distribution of the *E. coli*‐PPI‐Network was used to produce RandomNet‐SameDD. In total, 100 RandomNet‐SameDD for each *E. coli*‐PPI‐Network (constructed from overlapped interactions or single datasets) were produced for statistical analysis.

### Calculation of the degree of involved 1‐cycles of each protein (1‐DC_node_)

Because the *E. coli*‐PPI‐Network contained 1‐cycles, but very few 2‐cycles, we focused our analysis on the 1‐DC_protein_. First, all of the cycles during algebraic topology analysis were listed. Then, the degree of each node was calculated, which is denoted as *D*_protein_. Similarly, the number of *q*‐cycles containing a protein was defined as number of *q*‐cycles involving a protein (*q*‐DC_protein_), and the *q*‐DC_protein_ for each protein was calculated by examining all of the *q*‐cycles. Next, a rank value for a *q*‐cycle representing its importance was defined as the average value of all *q*‐DC_protein_ for each node in the *q*‐cycle. Then, the cycles can be ordered by their rank values representing their relative importance in the network.

### Calculation of traditional graph parameters of the network

Traditional graph parameters of the *E. coli*‐PPI‐Network were calculated as follows. (1) Degree: the number of neighbors of a node. (2) Cluster coefficient: for a node *n_i_* whose degree is *k_i_* (has *k_i_* neighbors), if the *k_i_* neighbors have *e_i_* edges, then the cluster coefficient for the node *n_i_* is the ratio of *e_i_* to all possible edges for the *k_i_* neighbors: cc*_k_* = (2*e_i_*)/(*k_i_* (*k_i_* − 1)). (3) Betweenness: the ratio of the number of the shortest path including a node (*s_i_*) to all possible shortest paths in the network: *b_k_* = (2*s_i_*)/((*N* − 1)(*N* − 2)) (here, *N* is the total number of nods in the network). (4) Closeness: the closeness of a node is defined as the sum of the multiplicative inverse of the shortest path to other nodes, and normalized by dividing (*N* − 1). The multiplicative inverse is used to avoid the situations of infinite values of shortest path. (5) Assortativity for degree, closeness, and betweenness: the assortativity of a node is the coefficient between the distance and corresponding parameters (degree, closeness, and betweenness) to the other nodes. Similarly, the multiplicative inverse of the shortest path is used to avoid infinite values.

## Results and Discussion

### Construction of a statistically significant reliable PPI network of *E. coli*

To get an accurate PPI network is still a challenging task despite advances in high‐through technologies. Therefore, we first used novel statistical tools based on GHGD [19] to produce a statistically reliable combined PPI network from several datasets. In the present study, three datasets from independent research groups were used, those of: Arifuzzaman *et al*. [17], Hu *et al*. [18], and Rajagopala *et al*. [21]. There are 11 017, 3888, and 5993 interactions in the three datasets, respectively, as well as a total of 3485 nodes (proteins) and 19 719 interactions (Table S1). Examination of the PPI overlaps in the three datasets revealed that there are only 37 interactions that were overlapped in all of the three datasets (PPI(OL = 3)), whereas there are 1142 interactions that were overlapped in at least two datasets (PPI(OL ≥ 2)) (Table S1).

Notably, only a small number of interactions (total of 37) were overlapped in all three datasets. Therefore, a prominent question is how to use the data in the three datasets to get a combined PPI network. In detail, if we use the (PPI(OL = 3)), we would loss too many edges (high false negative), whereas, if we use the (PPI(OL ≥ 1)), we would get too many false positives. Because there are 3485 proteins (nodes) in the three datasets, there are a total of 3485 × (3485 − 1)/2 = 6 070 870 potential interactions among these nodes. The question is: by selecting three subsets (containing 11 017, 3888, and 5993 interactions, respectively) among these potential ones (6 070 870), what is the probability that there are 1142 interactions overlapped in at least two subsets? By using the GHGD [19], we found that the 95% CI of number of PPI(OL ≥ 2) when randomly selected was 0.94–42.57 (Table 1), indicating that, at a statistical level of 0.05, there were at most 42 interactions in the 1142 PPI(OL ≥ 2) which may not be statistically significant (false positive is about 3.68%) (Table 1). The GHGD analysis demonstrated that the identified 1142 interactions (PPI(OL ≥ 2)) were highly reliable and were used to construct a statistically reliable *E. coli*‐PPI‐Network.

**Table 1:** General hypergeometric distribution analysis of the overlapped interactions in the three datasets. NOL, number of elements with specific overlapped feature.

Total potential amount	Subsets selected	Number of independent groups	Overlap number	Number of overlapped genes	Mean of NOL distribution	Var of OL distribution	95% CI of NOL distribution	False positive (*P* < 0.05)
6 070 870	11 017, 3888, 5993	3	≥ 3	37	0.007	0.007	0–0.38	0.00%
6 070 870	11 017, 3888, 5993	3	≥ 2	1142	21.76	21.66	0.94–42.57	< 3.68%

### *E. coli*‐PPI‐Network has special HDATS

As described above in methods (Calculation of homology group (HG) and Betti numbers) [15], we analyzed the algebraic topological structures of the *E. coli*‐PPI‐Network by using our established program based on jplex. Different from traditional graph theory, algebraic topology further studied the intrinsic properties of the global structures of the graphs or networks. Remarkably, homology group (HG) *H*_q_(*K*) (here *q* is the dimension of simplex) of the simplicial complex, a common conception in modern algebraic mathematics, was introduced to quantitatively describe the nature of the network in a precise manner. The definition of simplex, cycle, HG, and calculation of the HG were described in detail in our previous paper and in the Methods section. The most important parameter of the HG is *q*‐dimension Betti numbers (Betti_q_) (here *q* is dimension; for detailed information, see Methods), which is defined as the rank of the homology group in each dimension. Intuitively, Betti numbers of the HG indicate the number of ‘holes’ or ‘cavities’ in each dimension. Intuitively, the betti_1_ represents the number of 1‐dimentional cavities (1‐cavity) in a graph, whereas Betti_2_ represents the number of 2‐dimentional cavities. Noted that *q*‐cavities are enclosed by *q*‐cycles, where *q*‐cycles are *q*‐chains that are closed. The 1‐cycles can be viewed as traditional rings in a graph, whereas 2‐cycles can be viewed as the surface of hollow spherical structures. Similarly, for dim > 2, the Betti_q_ reflects the number of *q*‐dimension cavities that were enclosed by *q*‐cycles. From the definition, it should be noted that Betti numbers represent the amount of equivalence classes of cycles enclosing the cavities. Therefore, there would be many cycles that are equivalent to each other that surrounding a same ‘cavity’.

First, we examined the degree distribution of the network. The degree of a node in a network is defined as number of edges (or neighbors) that the node has. As a result, all the nodes’ degree sequence has a power‐law distribution (Fig. S1).

Next, we examined the amount of simplexes in the network. Here, a *q*‐dimensional simplex (*q*‐simplex) in a network is defined as (*q* + 1) nodes that are connected to each other. For example, a vertex itself is a 0‐simplex, an edge with two connected points is a 1‐simplex, a triangle composed of three points connected to each other is a 2‐simplex, and so on. As a result, the *E. coli*‐PPI‐Network had a biggest dimension of simplex (BDS) of 5 (the maximal dimension for all simplex) and contained 163 2‐simplexes, 53 3‐simplexes, 13 4‐simplexes, and two 5‐simplexes (Table S2).

Another important parameter is maximal simplex. First, a proper subtest of a simplex is a ‘face’ of the simplex. Therefore, a maximal simplex (max‐simplex) is a simplex that is not a face of any other simplexes in a network. We next analyzed the max‐simplexes of the *E. coli*‐PPI‐Network. Obviously, the BDS of max‐simples is the same as simplex. As a result, the *E. coli*‐PPI‐Network contained 58 2‐max‐simplexes, 18 3‐max‐simplexes, two 4‐max‐simplexes, and two 5‐max‐simplexes (Table S3).

Furthermore, we analyzed the cavities of the *E. coli*‐PPI‐Network by calculating HGs. As a result, the *E. coli*‐PPI‐Network had the biggest dimension of cavity (BDC, the maximal dimension for all cavities) of 2 and contained a total of 149 0‐dimensional cavities (0‐cavity), 79 1‐cavities, and one 2‐cavity (Table 2). It should be noted that the 0‐cavity indicates the number of disconnected subnetworks of a network. For the 79 1‐cavities, there are 79 classes of equivalent cycles, which surrounded 79 cavities. For the one 2‐cavity, there is a 2‐cavity in the network, which is enclosed by an equivalent class of 2‐cycles.

**Table 2:** Number of cavities (Betti numbers) in different dimensions in *Escherichia* *coli*‐PPI‐Network, control RandomNet and RandomNet‐SameDD.

Dimension	*E. coli*‐PPI‐Network	RandomNet			RandomNet‐SameDD		
		Average	SD	95% CI	Average	SD	95% CI
0	149	160.00	10.22	139.96–180.04	315.20	4.19	306.99–323.41
1	79	208.20	10.21	188.19–228.21	31.65	3.66	24.48–38.82
2	1	0.00	0.00	0.00–0.00	0.00	0.00	0.00–0.00

### The HDATS of the *E. coli*‐PPI‐Network are significantly different from random networks

**Fig. 2:**
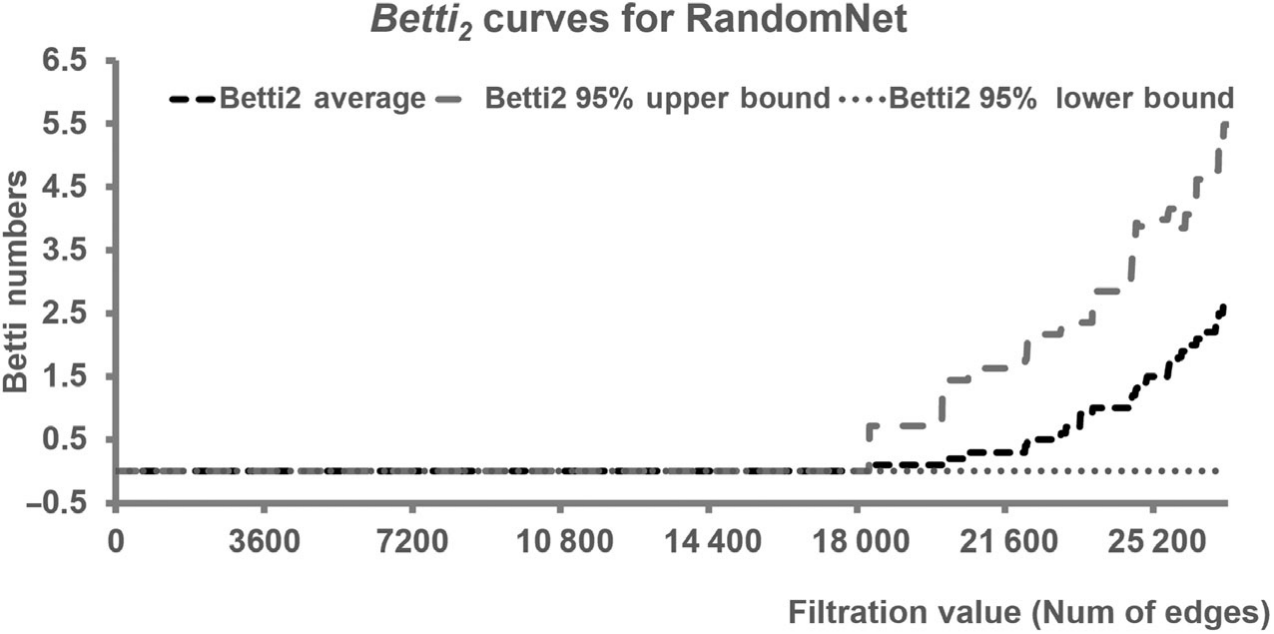
Betti curves of dimension 2 in RandomNets, showing the number of 2‐cavities during increased number of edges.

We have demonstrated that the *E. coli*‐PPI‐Network had remarkable HDATS. However, any network has its own algebraic topological structures. Therefore, it is necessary to determine whether these HDATS are just random noises or have specific meanings. To this end, we produced random networks with the same number of vertex and edges (RandomNet). In total, 1000 randomNets with the same number of vertex and edges were produced, and their corresponding algebraic topological parameters were calculated, including amount of simplexes, max‐simplexes, and cavities in each dimension. By analyzing the distribution of these parameters of the randomNet, we found that randomNet had much smaller BDS and BDC, which formed much lesser high‐dimensional simplexes and max‐simplexes. For example, there are a total of 165 2‐simplexes and 58 2‐max‐simplex in the *E. coli*‐PPI‐Network, but few, if any, 2‐simplexes were produced in the RandomNet (Table S2). Notably, the BDC for RandomNets is 1 and no cavities with dimension ≥ 2 were produced (Table 2). In addition, by investigating the random networks, especially the Betti curves of Betti_2_ versus number of edges, we found that the 2‐cavities can be formed when the number of edges reaches approximately 18 000 (Fig. 2), whereas the *E. coli*‐PPI‐Network only has 1142 edges, indicating that the formation of a 2‐cavity is extremely specific for the *E. coli*‐PPI‐Network.

### The HDATS of the *E. coli*‐PPI‐Network are not produced by special random networks with the same degree distribution

A key topological feature of biological networks is scale‐free property, as indicated by their paw law distributions of degrees, which would influence the structures of the network remarkably. We next examined whether the HDATS observed in *E. coli*‐PPI‐Network can be produced by its scale‐free property. To this end, we constructed random networks with the exactly same degree distribution (RandomNet‐SameDD) of the *E. coli*‐PPI‐Network. The RandomNet‐SameDD were constructed based on the Havel–Hakimi theorem. Similarly, 1000 random RandomNet‐SameDD were produced and analyzed. Remarkably, the BDS in RandomNet‐SameDD was 9, which is much higher than that of *E. coli*‐PPI‐Network for which the BDS is 5. However, even with much higher BDS and more high‐dimensional simplexes (Table S2), the RandomNet‐SameDD exhibited much less 1‐cavities, and, remarkably, no 2‐cavities (Table 2). In addition, the RandomNet‐SameDD had more 0‐cavities. The larger value of Betti_0_ and more high‐dimensional simplexes indicated that, compared with the *E. coli*‐PPI‐Network, these RandomNet‐SameDD tend to be more aggregated locally but separated globally. Overall, the HDATS observed in *E. coli*‐PPI‐Network are not produced by its scale‐free property and would have specific significance.

### HDATS were also observed in PPI‐Networks constructed from an individual dataset

We next examined whether the HDATS were also observed in the networks constructed from an individual dataset, to exclude the possibility that the process of using overlapped interactions would bring biases. The networks constructed from the three datasets [Arifuzzaman *et al*. [17], Hu *et al*. [18], and Rajagopala *et al*. [21]] were labeled as *E. coli*‐PPI‐Network‐A, *E. coli*‐PPI‐Network‐P, and *E. coli*‐PPI‐Network‐R, respectively. As a result, all of the three *E. coli*‐PPI‐Networks had high‐dimensional simplexes (BDS 6–8), and, remarkably, all of the networks have 2‐cavities (Table 3), and one had a 3‐cavity. In detail, compared with the RandomNet, all of the individual *E. coli*‐PPI‐Network had more simplexes and cavities for dimensions ≥ 2. Compared with their corresponding RandomNet‐SameDD, all of the individual *E. coli*‐PPI‐Network had smaller BDS and less simplexes in high dimensions (≥ 2), and, in contrast, had more cavities in high dimensions (≥ 2). All of these results were consistent with that of the combined *E. coli*‐PPI‐Network.

**Table 3:** Key algebraic topological parameters of individual *Escherichia* *coli*‐PPI‐Network and corresponding RandomNetand RandomNet‐SameDD. Rand, RandomNet; Rand SameDD, RandomNet‐SameDD. For RandomNet and RandomNet‐SameDD, 200 samples were produced to get the results. The average values of the random networks are shown.

Network	BDS			BDC			Betti2		
	*E. coli*	Rand	Rand SameDD	*E. coli*	Rand	Rand SameDD	*E. coli*	Rand	Rand SameDD
*E. coli*‐PPI‐Network‐A	6	2	34	2	1	1	48.00	0.00	0.00
*E. coli*‐PPI‐Network‐P	6	2	18	2	1	1	1.00	0.00	0.00
*E. coli*‐PPI‐Network‐R	8	3	36	3	2	1	192.00	31.10	0.00
*E. coli*‐PPI‐Network	5	2	9	2	1	1	1.00	0.00	0.00

**Fig. 3:**
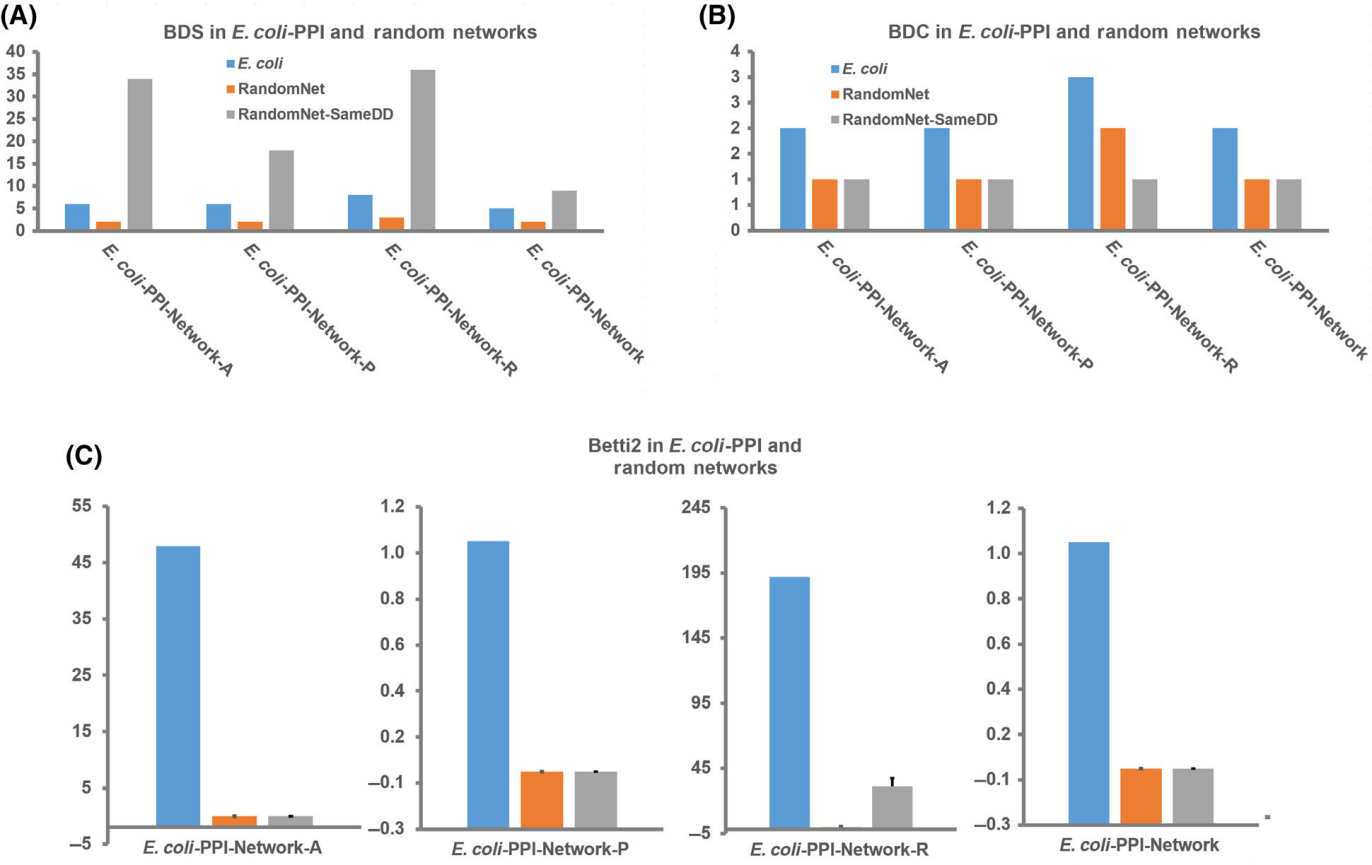
Key parameters of the *Escherichia coli*‐PPI‐Networks constructed from individual or combined datasets, including (A) BDS, (B) BDC, and (C) Betti2 values. For the RandomNet and RandomNet‐SameDD, error bars represent the SD and *n* = 100.

Taken together, these results demonstrated that *E. coli*‐PPI‐Network had less high‐dimensional simplexes but more high‐dimensional cavities (Fig. 3 and Table 3). Intuitively, from a geometric view, nodes in the *E. coli*‐PPI‐Network did not connect to each other as dense as in the RandomNet‐SameDD and formed many high‐dimensional cavities. The special algebraic topological structures of the *E. coli*‐PPI‐Network with various cycles enclosing the cavities may reflect certain uncovered intrinsic natures of the roles of information flow in the whole network, which is not clear and needs further investigation.

### Analysis of cycles of the *E. coli*‐PPI‐Network revealed potential key molecules underlying the biological dynamics of *E. coli*

For traditional graph analysis of PPI networks, nodes with high degree (or hubs) are often considered to be important for the network. Because degree is a local parameter related only to the neighbors of a node, much potential information related to the integrative features of the network would be lost [22], especially those related to high‐dimensional cycles, which enclosed cavities. Therefore, based on our above results, we further analyzed the cycle‐related features of each node.

First, we analyzed all the components of 1‐cycles in the *E. coli*‐PPI‐Network. As revealed above, there are a total of 79 1‐cavities in the *E. coli*‐PPI‐Network. We first defined the degree of involved *q*‐cycles of a node as:

$$q‐{\mathrm{DC}}_{\mathrm{protein}}=\mathrm{the}\;\mathrm{number}\;\mathrm{of}\;q\text{-cycles}\;\mathrm{that}\;a\;\mathrm{node}\;\mathrm{is}\;\mathrm{involved}\;\mathrm{in}\;\mathrm{the}\;\mathrm{network}.$$

**Fig. 4:**
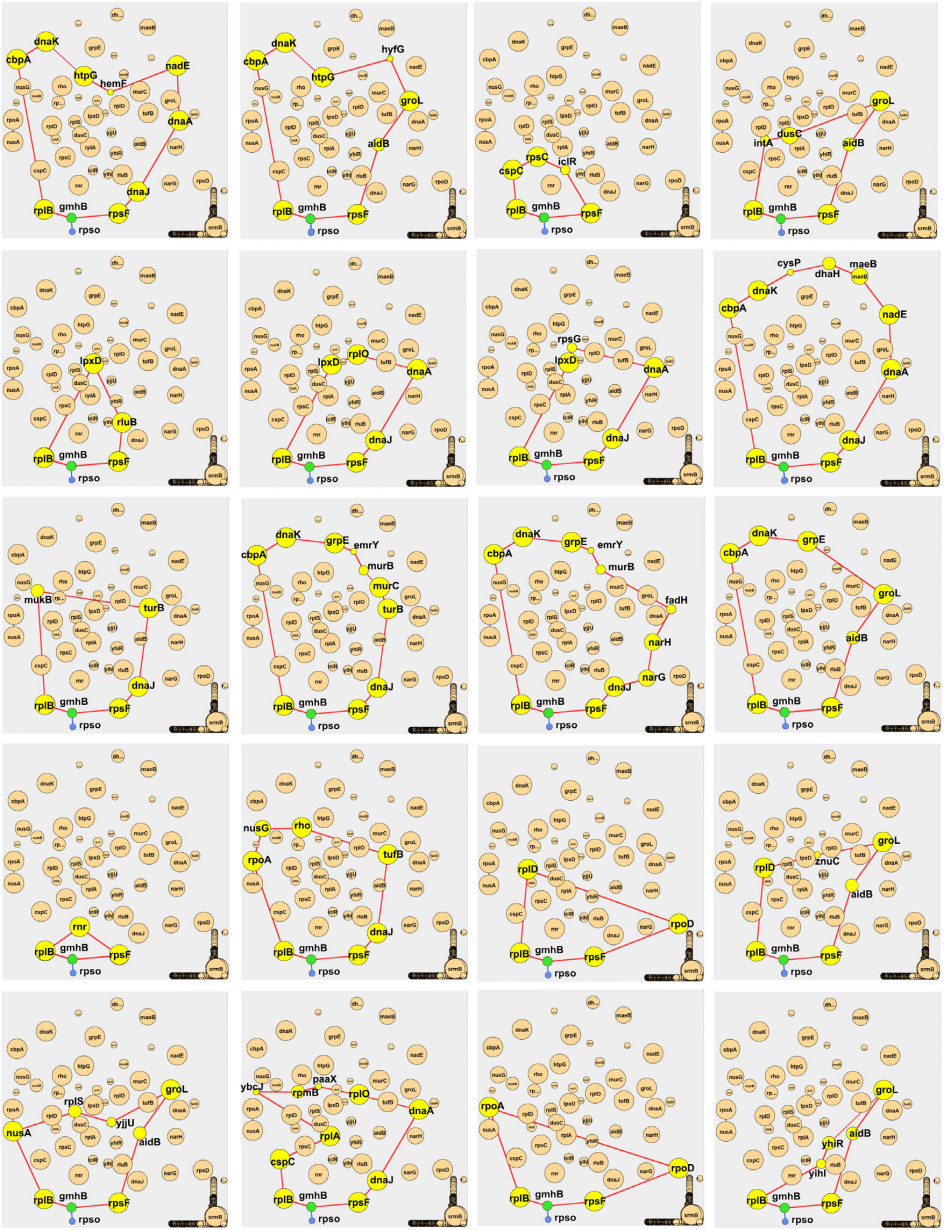
Representative gene node gmhB, who had low degree value (3), but high 1‐DC_protein_ (20). All of the 20 1‐cycles for which the gmhB involved are shown, as well as all of its neighbors (rplB, rpsF, rpso). Yellow nodes: node genes that form one of the cycles involving gmhB. Green node: node gene for gmhB. Blue nodes: node genes which are not involved in the cycles involving gmhB. The size of the node represents the degree of the genes (larger nodes have greater degree values).

Therefore, different from traditional degree, *q*‐DC_protein_ further described the number of cycles that a protein participates. We analyzed the 1‐DC_protein_ for all of the nodes (Table S4). The top 15 proteins ranked by 1‐DC_protein_ are: rplB, rpsF, yfgB, rluB, rplD, tufB, ybjX, dnaJ, rnr, groL, aidB, dnaA, gmhB, selB, and cspC. Interestingly, some of the proteins had relatively small degrees, such as ybjX, aidB, dnaA, gmhB, and cspC (whose degrees were between 3 and 7). For example, gmhB has a degree of 3, but a 1‐DC_protein_ value of 20 (Fig. 4). In addition, the node groL had the largest degree, but not the largest 1‐DC_protein_. These results indicated that the algebraic topological analysis revealed additional novel features of the proteins in the network. Most of the proteins are related to the critical biological processes of the bacteria such as ribosomal subunit, RNase, protein translation, lipid biosynthesis, and drug resistance (Table S4), which constitute the basic components of bacteria survival.

We also performed Gene Ontology (GO) enrichment to analyze the molecular functions of the genes comprising the cycles. To this, GO enrichment of the genes in each 1‐cycle in the *E. coli*‐PPI‐Network was performed, and the top five GO items with *P* < 0.05 for each cycle were obtained (Table S5). Next, the frequencies of the GO items in the top five items in all 1‐cycles were ranked. As a result, the most frequent items of enriched molecular functions are involved in structural constituent of ribosome, RNA binding, protein binding, DNA replication, translation, and so on. (Table S6). In addition, from the GO enrichment analysis in each 1‐cycle, we also identified subcycles that are involved in certain molecular functions. For example, in a cycle (aidB‐>groL‐>helD‐>pepB‐>rpoD‐>rplD‐>cbpA‐>rplB‐>gmhB‐>rpsF‐>aidB), there is a subcycle containing three genes (rplB, rplD, and rpsF), which are significantly involved in structural constituent of ribosome (GO:0003735, *P* = 1.13 × 10^−4^) (Table S5). These results implied that the 1‐cycles identified in the *E. coli*‐PPI‐Network are involved or related to many key programs of the bacteria.

### HDATS revealed novel node features of the PPI‐Networks which are different from traditional graph analysis

**Fig. 5:**
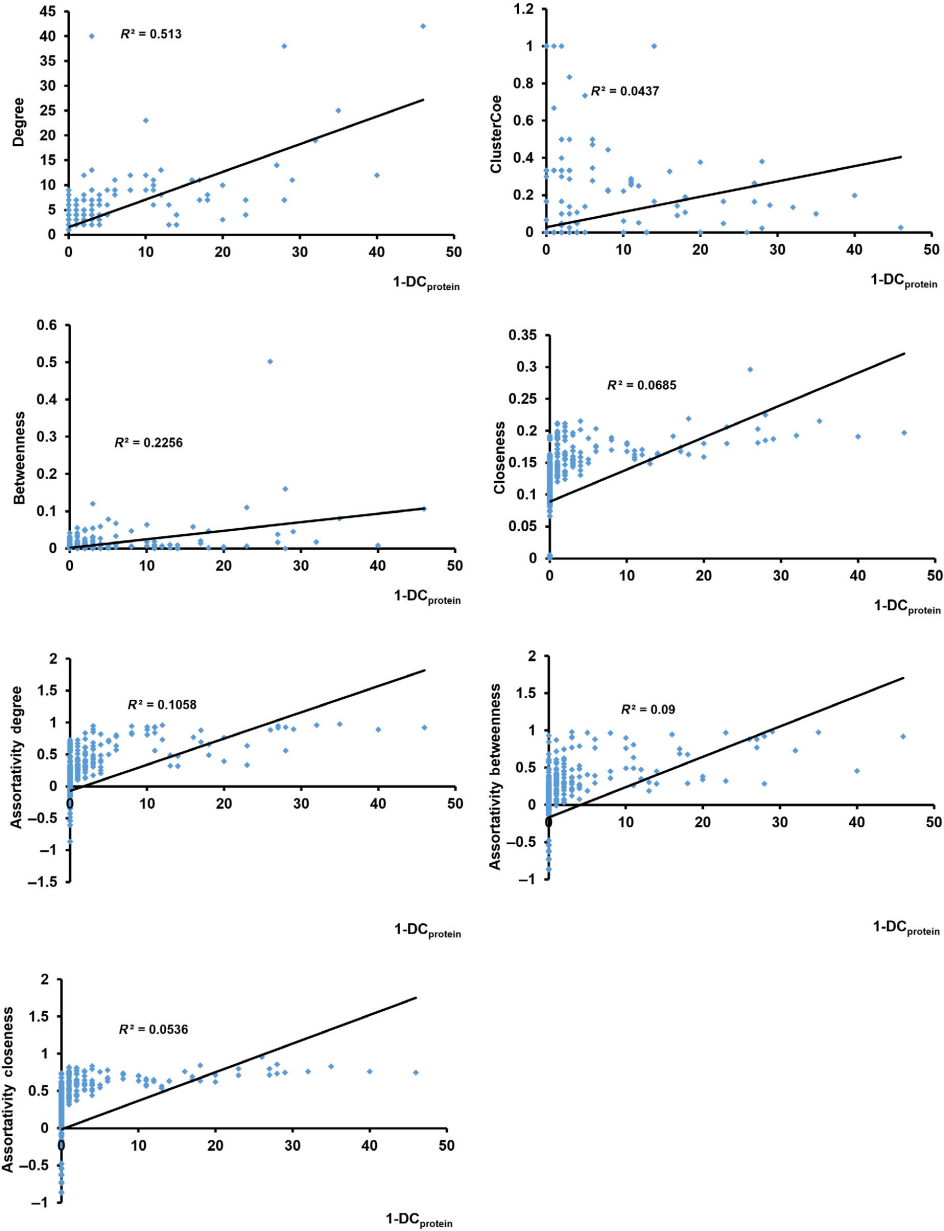
Correlation between 1‐DC_protein_ and traditional parameters, including degree, cluster coefficient, betweenness, assortativity (assortativity of degree, closeness, and betweenness).

We have addressed that *E. coli*‐PPI‐Network exhibited special algebraic topological structures. Especially, the nodes exhibited novel features, such as *q*‐DC_protein_, which is different from that of traditional parameter. Therefore, we further investigated the relationship and difference between *q*‐DC_protein_ and traditional parameters, such as degree, cluster coefficient, betweenness, and assortativity (assortativity of degree, closeness, and betweenness) (Table S4). In the combined *E. coli*‐PPI‐Network, correlation analysis revealed that the 1‐DC_protein_ had a strong positive correlation with the degree (*R* ^2^ = 0.51) and weak correlation with the other parameters (Fig. 5). Nevertheless, there is also apparent difference between these two features. As described above, there are genes who had large 1‐DC_protein_ value but relatively small degrees (Table S4). Similarly, there are also genes that had large degree values but relatively low 1‐DC_protein_ values. These data indicated that 1‐DC_protein_ reflected novel topological features of the network that cannot be fully described by traditional graph features.

## Conclusions

In recent years, biological network construction and analysis has been an important approach for identifying potential drug targets in various situations, such as in cancer [15], infective diseases (especially for multidrug resistance bacteria) [23], and COVID‐19 [24, 25]. For example, by analyzing the essential genes in the COVID‐19‐related biological networks, candidates as potential COVID‐19 treatments were identified [24, 25]. Therefore, network analysis played an important role in revealing the rules of biological processes and identifying potential treatment targets. Traditional graph analysis provided a hand of tools for characterizing the features of a network such as degree, cluster coefficient, betweenness, assortativity, and so on. However, these features lack a characterization of the integral and especially high‐dimensional features of the network, such as high‐dimensional cavities or cycles. In the present study, by introducing algebraic topology, we studied the HDATS and found that the *E. coli*‐PPI‐Network had special HDATS that are significantly different from random networks. Notably, these special HDATS cannot be produced by random networks, especially the random one with the same degree distribution, indicting that HDATS uncovered novel network features different from traditional network characteristics such as small world features. In addition, based on the definition of cycles in the network, we also expanded the concept of ‘degree’ in the traditional graph analysis; that is, by introducing *q*‐DC_protein_, we also investigated the number of *q*‐dimensional cycles that a node (protein) anticipates. It should be noted that the definition of *q*‐DC_protein_ is a novel concept based on a perspective of integrative topological features, which is different from traditional parameters such as hub or node degree. Indeed, hub features are not sufficient for completely describing the properties of a network [22]. Furthermore, the present approaches and results can be used to identify potential therapeutic strategies for diseases, such as developing novel types of antibiotics and overcoming the drug resistance of bacteria.

There are also limitations to the present study. In algebraic topology calculation, based on the definition of the quotient group, a ‘cycle’ represents a class of equivalent cycles, which enclose the same cavity. The description and analyses of a class of equivalent cycles with more accuracy will be the subject of our future studies.

In conclusion, by taking advantage of GHGD distribution, we reconstructed a statistically reliable combined *E. coli*‐PPI‐Network. From an algebraic topological view, we discovered novel HDATS properties of the *E. coli*‐PPI‐Network, which cannot be obtained by traditional graph analysis. We further defined new features of a node, which is *q*‐DC_protein_ in a network, a concept based on the algebraic topological features of a network, and greatly extended the characteristics of a node beside traditional parameters. Our study revealed potential rules of information flow in *E. coli*, which would have implications for identifying the mechanisms of key processes of bacteria such as survival, drug resistance, and mechanisms of diseases in human.

## Conflict of interest

The authors declare no conflict of interest.

## Author contributions

XX and XM conceived the experiments. ZC, YH, and DN performed the experiments, collected data, and performed part of the data analysis. HZ, XX, and XM performed the data analysis. XX and XM co‐wrote the paper.

## Supporting information


**Fig. S1.** Degree distributions (or degree sequence) of the *E. coli*‐PPI‐Network.
**Table S1.** Parameters of the networks provided in three different research groups.
**Table S2.** Number of simplexes with different dimensions in *E. coli*‐PPI‐Network and control RandomNet and RandomNet‐SameDD.
**Table S3.** Number of maximal simplexes with different dimensions in *E. coli*‐PPI‐Network and control RandomNetand RandomNet‐SameDD.

**Table S4.** 1‐DC_protein_, and traditional graph parameters of the *E. coli*‐PPI‐Network.

**Table S5.** 1‐cycles and the top five GO enrichment items for the genes of the cycle.
**Table S6.** Ranked enriched GO items (molecular functions) in 1‐cycles in the *E. coli*‐PPI‐Network.

## Data Availability

The data that support the findings of the present study, as well as the code, are available in: Mao, Xing‐gang (2022), ‘Algebraic Topological Analysis of networks’, Mendeley Data, V1, https://doi.org/10.17632/3rj8rsbmhd.1 (https://data.mendeley.com/datasets/3rj8rsbmhd/1).
