# Supplementary material for: Protein–protein interaction network of E. coli K‐12 has significant high‐dimensional cavities: new insights from algebraic topological studies
Source: FEBS Open Bio. 2022 Jun 16;12(7):1406–18. doi: 10.1002/2211-5463.13437 (PMC9249336; doi:10.1002/2211-5463.13437)
Supplement: Supplementary file 1 — Fig. S1. Degree distributions (or degree sequence) of the E. coli‐PPI‐Network. Table S1. Parameters of the networks provided in three different research groups. Table S2. Number of simplexes with different dimensions in E. coli‐PPI‐Network and control RandomNet and RandomNet‐SameDD. Table S3. Number of maximal simplexes with different dimensions in E. coli‐PPI‐Network and control RandomNetand RandomNet‐SameDD. [file FEB4-12-1406-s002.docx]

**Supplementary data**

**Supplementary Tables**

**Supplementary Table S1**

parameters of the networks provided in 3 different research groups

| Dataset | Number of nodes | Number of edges |
| --- | --- | --- |
| Arifuzzaman et al. | 2931 | 11017 |
| Pingzhao et al | 2039 | 3888 |
| Rajagopala, et al. | 1757 | 5993 |
| Overlapped in at least 1 datasets | 3485 | 19719 |
| Overlapped in at least 2 datasets | 2225 | 1142 |
| Overlapped in at least 3 datasets | 1017 | 37 |

**Supplementary Table S2**

. number of simplexes with different dimensions in *E.coli*-PPI-Network and control RandomNet　and RandomNet-SameDD

| dimension | *E.coli*-PPI-Network |  | RandomNet |  |  |  | RandomNet-SameDD |  |
| --- | --- | --- | --- | --- | --- | --- | --- | --- |
|  |  | Average | SD | 95%CI |  | Average | SD | 95%CI |
| 0 | 1092 | 1092.00 | 0.00 | [1092.00~1092.00] |  | 1092.00 | 33.05 | [1092.00~0.00] |
| 1 | 1142 | 1142.00 | 0.00 | [1142.00~1142.00] |  | 1142.00 | 33.79 | [1142.00~0.00] |
| 2 | 163 | 1.80 | 1.01 | [-0.17~3.77] |  | 734.82 | 26.68 | [757.73~136.65] |
| 3 | 53 | 0.00 | 0.00 | [0.00~0.00] |  | 852.54 | 28.15 | [912.52~936.49] |
| 4 | 13 | 0.00 | 0.00 | [0.00~0.00] |  | 797.79 | 26.47 | [895.08~2463.82] |
| 5 | 2 | 0.00 | 0.00 | [0.00~0.00] |  | 520.94 | 20.41 | [625.26~2833.03] |
| 6 |  |  |  |  |  | 228.48 | 12.47 | [301.57~1390.66] |
| 7 |  |  |  |  |  | 62.49 | 5.42 | [95.61~285.46] |
| 8 |  |  |  |  |  | 9.07 | 0.37 | [18.00~20.77] |
| 9 |  |  |  |  |  | 0.47 | 0.00 | [1.57~0.31] |

**Supplementary Table S3**

number of maximal simplexes with different dimensions in *E.coli*-PPI-Network and control RandomNet　and RandomNet-SameDD

| dimension | *E.coli*-PPI-Network |  | RandomNet |  |  |  | RandomNet-SameDD |  |
| --- | --- | --- | --- | --- | --- | --- | --- | --- |
|  |  | Average | SD | 95%CI |  | Average | SD | 95%CI |
| 0 | 0 | 134.55 | 10.23 | [114.50~154.60] |  | 0.00 | 0.00 | [0.00~0.00] |
| 1 | 936 | 1136.60 | 3.02 | [1130.69~1142.51] |  | 678.69 | 25.75 | [694.49~64.96] |
| 2 | 58 | 1.80 | 1.01 | [-0.17~3.77] |  | 114.29 | 10.00 | [128.67~53.82] |
| 3 | 18 | 0.00 | 0.00 | [0.00~0.00] |  | 22.70 | 3.85 | [30.54~16.01] |
| 4 | 2 | 0.00 | 0.00 | [0.00~0.00] |  | 11.52 | 2.27 | [17.89~10.58] |
| 5 | 2 | 0.00 | 0.00 | [0.00~0.00] |  | 12.99 | 2.66 | [18.92~9.14] |
| 6 |  |  |  |  |  | 4.94 | 1.29 | [8.21~2.78] |
| 7 |  |  |  |  |  | 5.60 | 1.39 | [9.26~3.49] |
| 8 |  |  |  |  |  | 4.37 | 1.17 | [7.38~2.36] |
| 9 |  |  |  |  |  | 0.47 | 0.00 | [1.57~0.31] |

**Supplementary Figures**


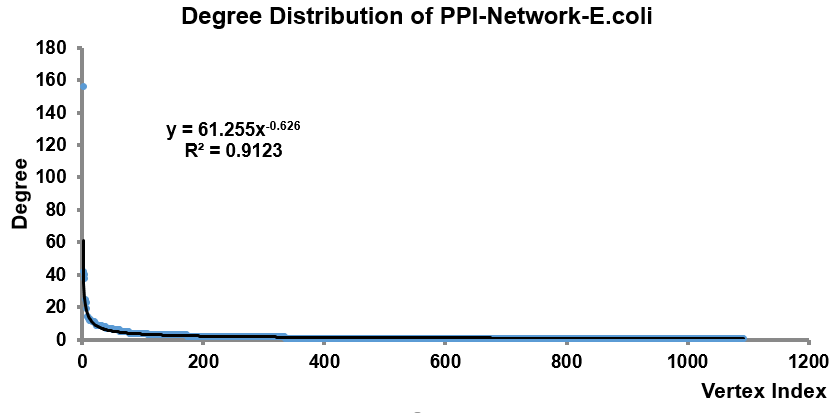


Figure S1. Degree distributions (or degree sequence) of the *E.coli*-PPI-Network.
